# Supplementary material for: A synergistic exploitation to produce high-voltage quasi-solid-state lithium metal batteries
Source: Nat Commun. 2021 Sep 30;12:5746. doi: 10.1038/s41467-021-26073-6 (PMC8484457; doi:10.1038/s41467-021-26073-6)
Supplement: Supplementary file 1 — Supplementary Information [file 41467_2021_26073_MOESM1_ESM.pdf]

Supplementary Information

A synergistic exploitation to produce high-voltage quasi-solid-state lithium metal batteries

Junru Wu[1,2], *Xianshu Wang*[1,2], *Qi Liu*[1,2], *Shuwei Wang*[1,2], *Dong Zhou*[1,2,3,*], *Feiyu Kang*[1,2], *Devaraj Shanmukaraj*[4], *Michel Armand*[4,*], *Teofilo Rojo*[5], *Baohua Li*[1,2,*], *Guoxiu Wang*[3,]

[1] Graduate School at Shenzhen, Tsinghua University, Shenzhen 518055, China

[2] School of Materials Science and Engineering, Tsinghua University, Beijing 100084, China

[3] Centre for Clean Energy Technology, School of Mathematical and Physical Sciences, University of Technology Sydney, Sydney, NSW 2007, Australia

[4] Centre for Cooperative Research on Alternative Energies (CIC energiGUNE), Basque Research and Technology Alliance (BRTA), Alava Technology Park, Albert Einstein 48, 01510 Vitoria-Gasteiz, Spain

[5] Inorganic Chemistry Department, University of the Basque Country UPV/EHU, Bilbao 48080, Spain

* Email: zhoudong087@gmail.com

marmand@cicenergigune.com

libh@sz.tsinghua.edu.cn

Guoxiu.Wang@uts.edu.au

Supplementary Figures

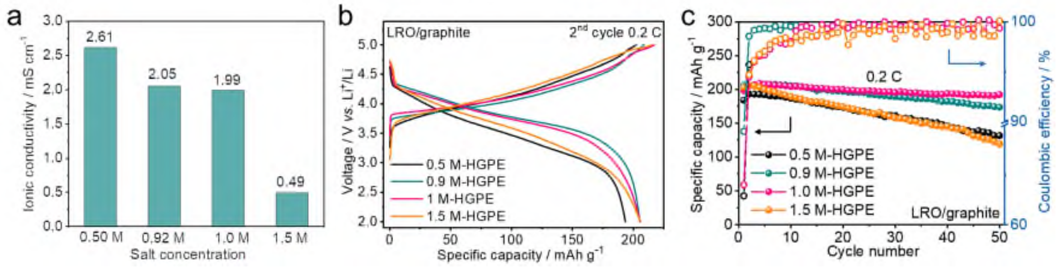

Supplementary Figure 1 **a** Ionic conductivities of HGPE with different salt concentrations at 25 ^{o}C. **b** Charge-discharge curves and **c** cyclic performances of Li||LRO/graphite cells using HGPEs with different salt concentrations.

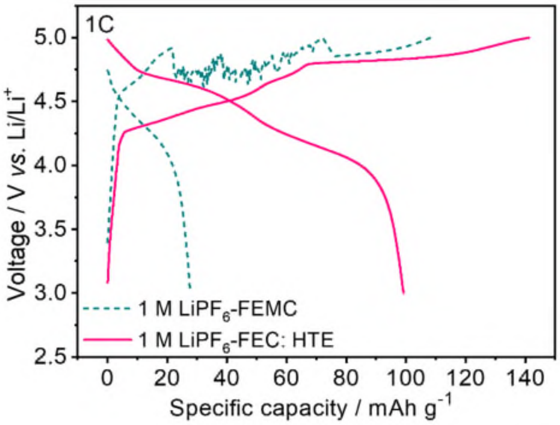

Supplementary Figure 2 Charge-discharge voltage profiles of a Li||KS6 graphite cell using different electrolytes at 2nd cycle.

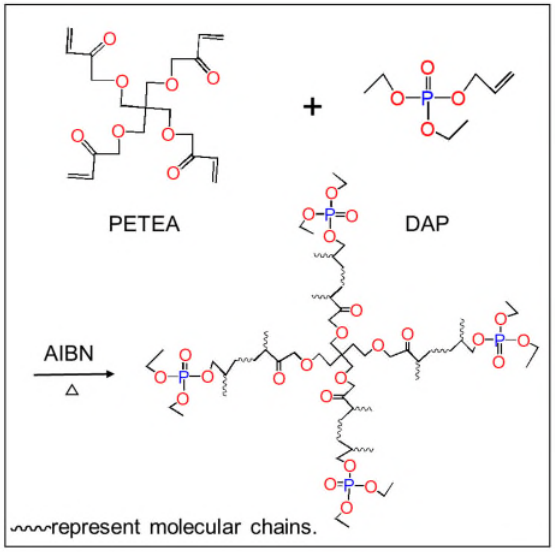

Supplementary Figure 3. *In situ* co-polymerization mechanism of DAP and PETEA monomers in liquid electrolyte.

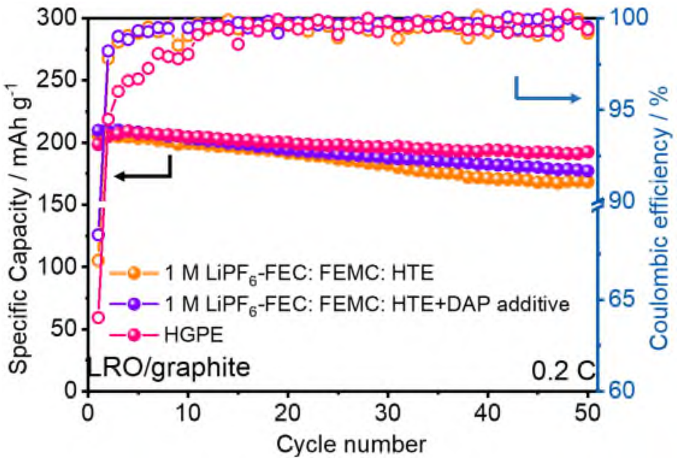

Supplementary Figure 4 Cyclic performances of Li||LRO/graphite cells using 1 M $LiPF_6$ in FEC: FEMC: HTE electrolyte without/with 2 wt% DAP additive and HGPE at 0.2 C. We have added 2 wt% DAP as electrolyte additive in the 1 M $LiPF_6$ in FEC: FEMC: HTE electrolyte. This concentration equaled to that of the residual DAP in the HGPE. As shown in Supplementary Figure 4, the addition of DAP obviously improved the capacity retention of Li|1 M $LiPF_6$ in FEC: FEMC: HTE|LRO/graphite cells (from 81.9 % to 84.8 % after 50 cycles) due to the SEI/CEI-forming ability of DAP. Moreover, the cell with HGPE showed higher cycling stability (96.9 % capacity retention after 50 cycles) than that of the cell using liquid electrolyte with DAP additive, because the polymer matrix inhibited the growth of Li dendrites.

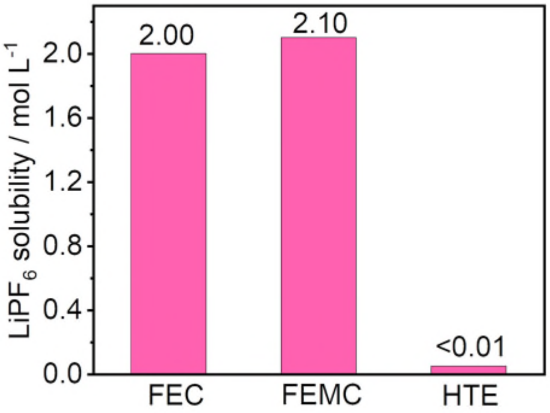

Supplementary Figure 5 The room temperature (25 ºC) solubility of $LiPF_6$ in FEC, FEMC and HTE solvents.

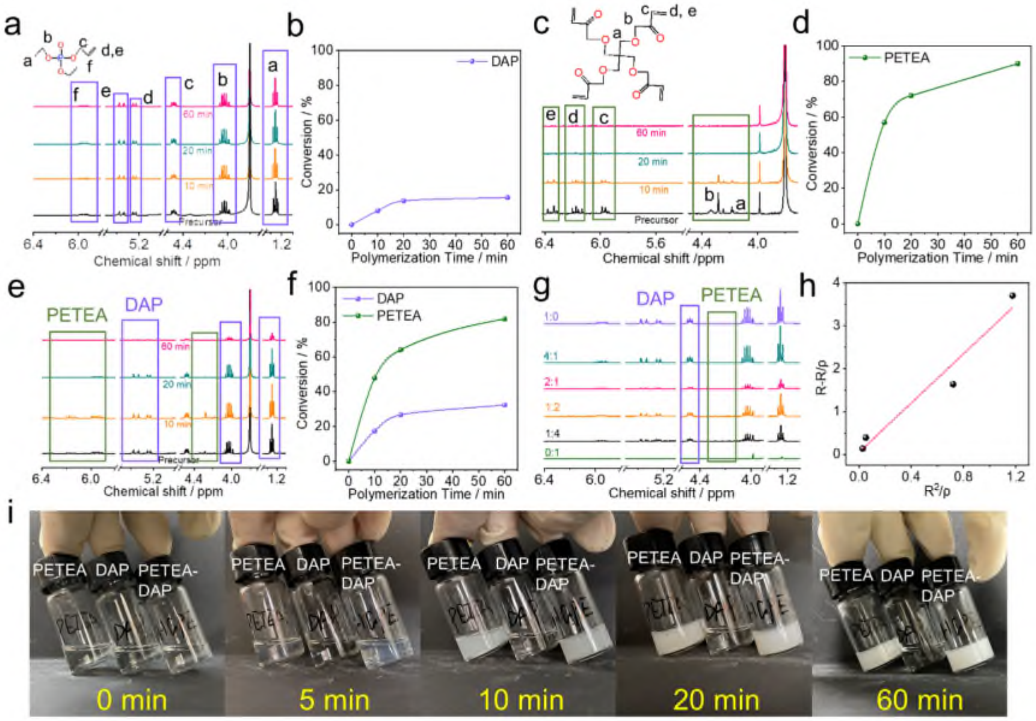

Supplementary Figure 6 **a**, **c**, **e** ^{1}H NMR spectra and **b**, **d**, **f** corresponding conversion rates of **a**, **b** 1.5 wt% PETEA only, **c**, **d** 3 wt% DAP only and **e**, **f** 1.5 wt% PETEA with 3 wt% DAP in 1 M $LiPF_6$-FEC: FEMC: HTE liquid electrolyte containing 0.1 wt% AIBN at different polymerization time. **g** ^{1}H NMR spectra of 4.5 wt% monomer in liquid electrolyte containing 0.1 wt% with different DAP: PETEA ratios after polymerizing at 70 ºC for 60 min. **h** The corresponding $(R-\frac{R}{\rho})-\frac{R^2}{\rho}$ plot. **i** The optical images of 1.5 wt% PETEA only (left), 3 wt% DAP only (middle) and 1.5 wt% PETEA with 3 wt% DAP (right) in liquid electrolyte containing 0.1 wt% AIBN at different polymerization time.

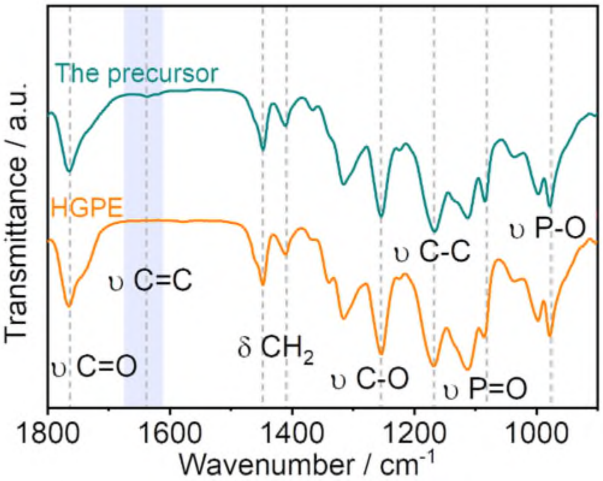

Supplementary Figure 7 FTIR spectra of the precursor solution and HGPE. The C=C at 1630 cm^{-1} almost disappear in the HGPE after polymerization, indicating a high conversion degree of monomers.

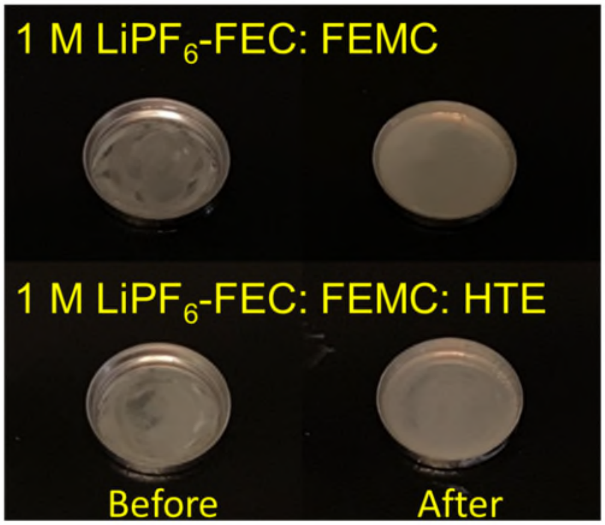

Supplementary Figure 8 Combustion tests of 1 M $LiPF_6$- FEC: FEMC (upper panels) and 1 M $LiPF_6$- FEC: FEMC: HTE (lower panels) electrolytes. The mass of each sample is set as 1 g.

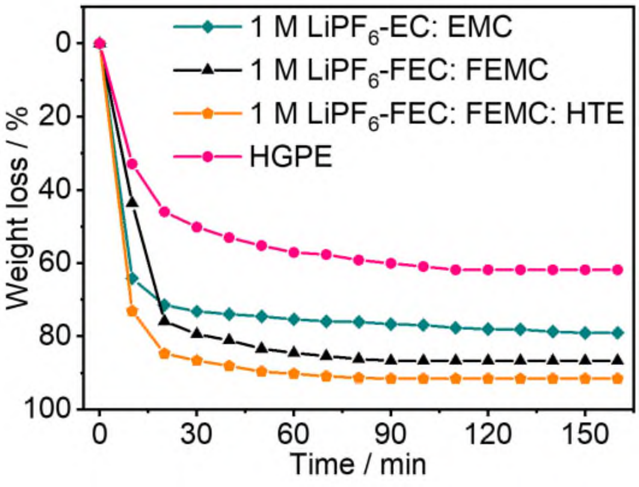

Supplementary Figure 9 Weight losses of the four electrolyte samples with aging time at 60 ºC.

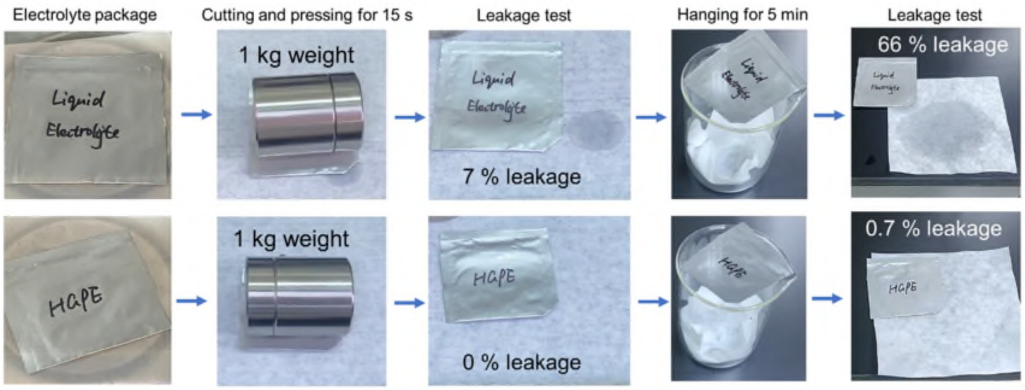

Supplementary Figure 10 Leakage tests of 1 M $LiPF_6$ in FEC: FEMC: HTE liquid electrolyte (upper panels) and HGPE (lower panels). we sealed 1 g 1 M $LiPF_6$ in FEC: FEMC: HTE liquid electrolyte and 1 g precursor solution of HGPE into Al plastic packages (55 mm × 50 mm), respectively, and heated the later to *in situ* form HGPE. Then a small notch was cut in each package, and then the packages were squeezed for 15 s under a 1 kg weight. The liquid electrolyte package showed a 6 wt% leakage, while HGPE was absolutely leak-free. Subsequently, the packages were further hung for 5 min. The weight loss was 66 % for the liquid electrolyte while that of HGPE was as low as 0.7 %, demonstrating the superior resistance of HGPE against liquid leakage.

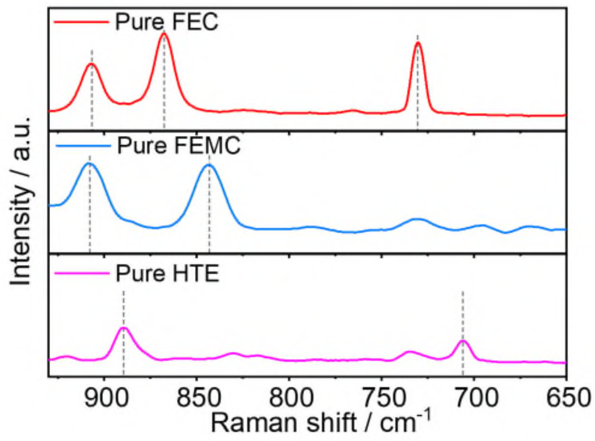

Supplementary Figure 11 Raman spectra of pure FEC, FEMC and HTE solvents.

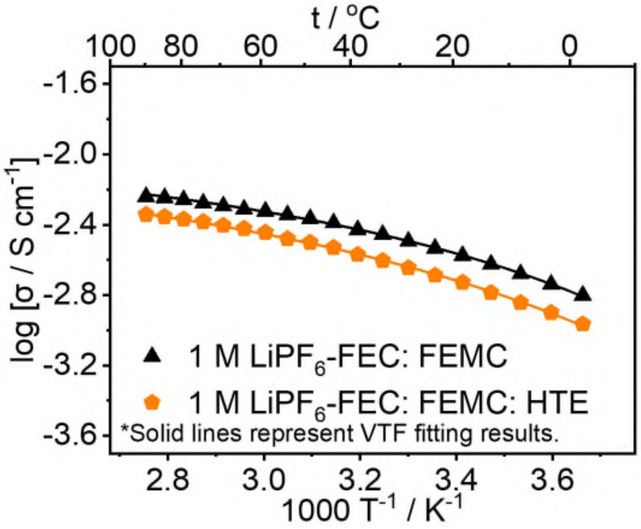

Supplementary Figure 12 Ionic conductivities for 1 M $LiPF_6$-FEC: FEMC and 1 M $LiPF_6$-FEC: FEMC: HTE at various temperatures from 0-90 °C. The plots represent the experimental data while the solid lines represent VTF fitting results.

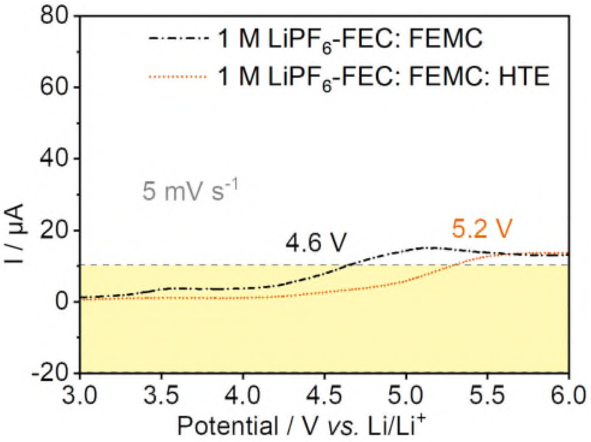

Supplementary Figure 13 LSV curves of the 1 M $LiPF_6$-FEC: FEMC and 1 M $LiPF_6$-FEC: FEMC: HTE electrolyte samples. The scan rate is 5 mV s^{-1}. Platinum foil are used as the working electrodes while Li foil is used as the counter and reference electrodes.

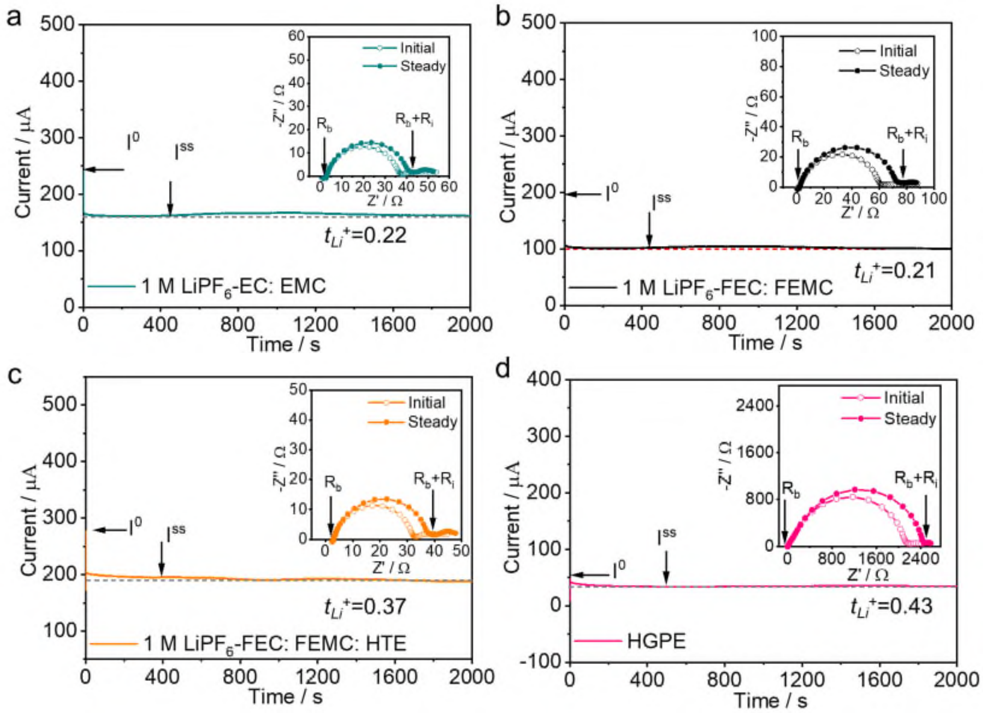

Supplementary Figure 14 The chronoamperometry profile of the symmetric Li||Li cells using different electrolytes. a Li|1 M $LiPF_6$-EC: EMC |Li cell; **b** Li|1 M $LiPF_6$-FEC: FEMC |Li cell; **c** Li|1 M $LiPF_6$-FEC: FEMC: HTE |Li cell and **d** Li|HGPE|Li cell. The applied polarization voltage is 10 mV. The EIS spectra before and after the polarization are shown in inset

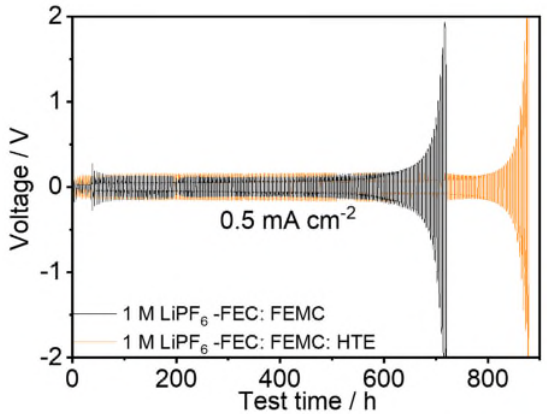

Supplementary Figure 15 Voltage profiles of the Li||Li symmetric cells using 1 M $LiPF_6$-FEC: FEMC and 1 M $LiPF_6$-FEC: FEMC: HTE electrolytes at 0.5 mA cm^{-2} with cut-off capacity of 1 mAh cm^{-2}.

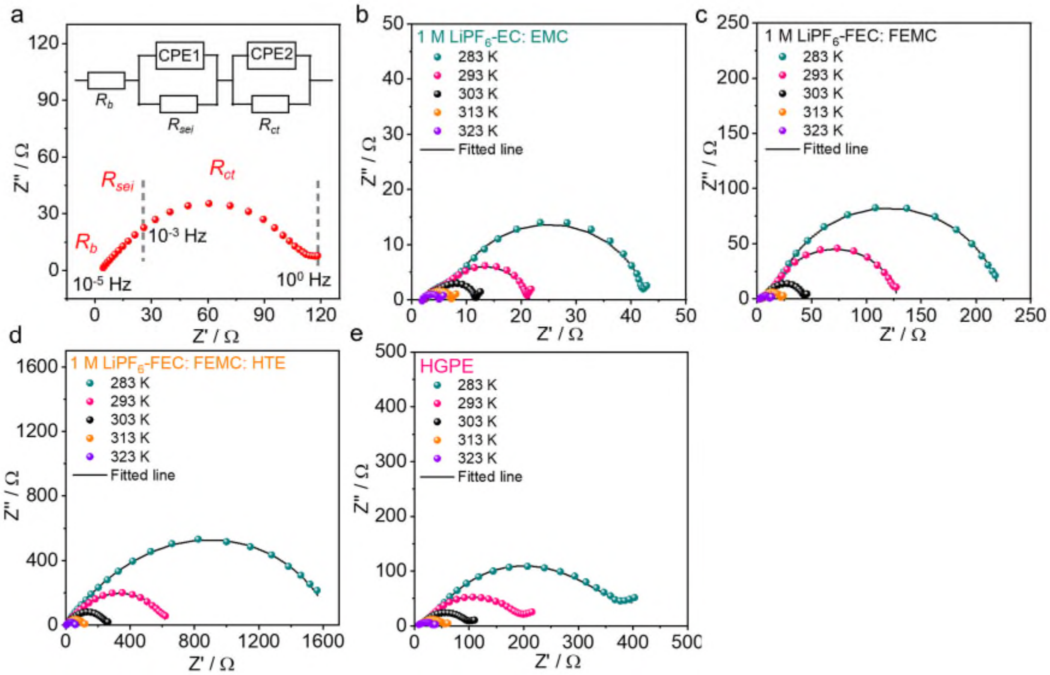

Supplementary Figure 16 Nyquist plots for Li||Li symmetric cells in the different electrolytes. a The equivalent circuit model and the corresponding Nyquist plot of Li||Li symmetric cells. R_b represents the bulk resistance, reflecting the resistance of electrodes, electrolyte, and separator. The semi-circle of R_{sei} in the high-middle frequency range represents the resistance of Li^+ transport through the SEI, while the semi-circle of R_{ct} in the low frequency range represents the de-solvation resistance of Li^+ before it enters the SEI. **b-e** Nyquist plots of cells using **b** 1 M $LiPF_6$-EC: EMC electrolyte, **c** 1 M $LiPF_6$-FEC: FEMC electrolyte, **d** 1 M $LiPF_6$-FEC: FEMC: HTE electrolyte and **e** HGPE at different temperatures. The errors between the raw and fitted EIS data were less than 2 %.

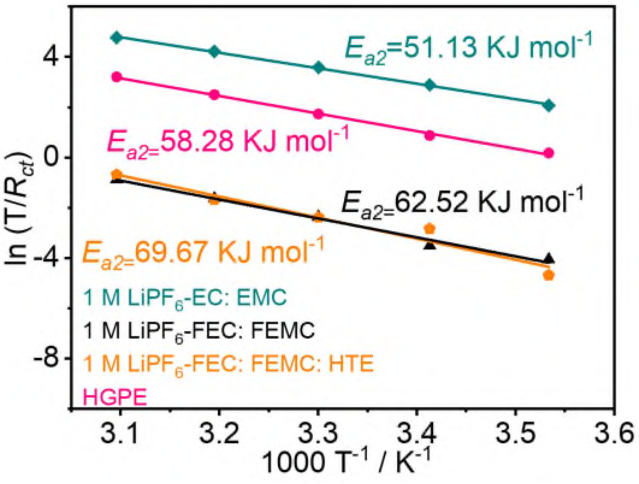

Supplementary Figure 17 The activation energies of R_{ct} derived from Nyquist plots.

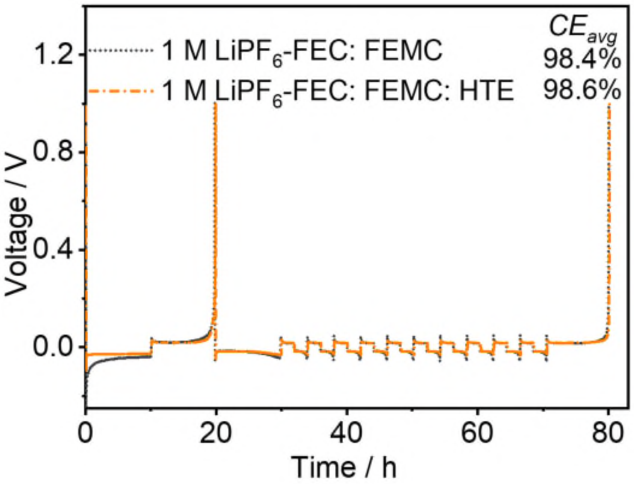

Supplementary Figure 18 CE_{avg} tests of Li plating-stripping in Li|1 M $LiPF_6$-FEC: FEMC|Cu and Li| 1 M $LiPF_6$-FEC: FEMC: HTE|Cu cells at 0.5 mA cm^{-2} with a capacity of 1 mAh cm^{-2}.

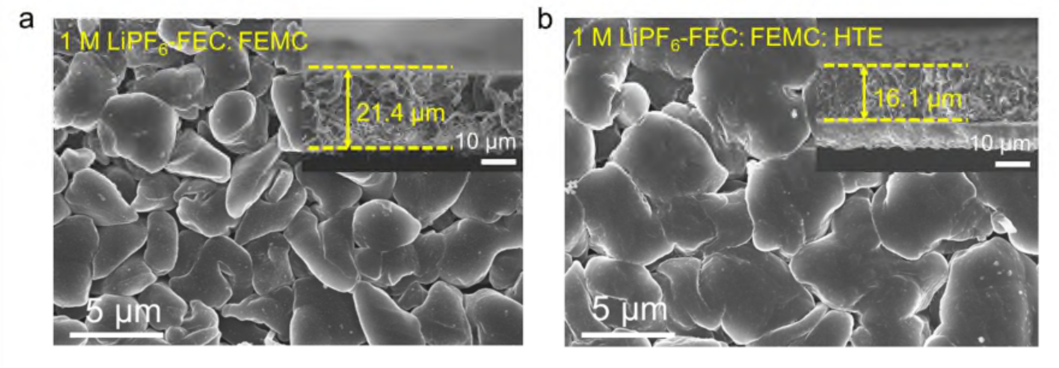

Supplementary Figure 19 Top and cross-sectional (shown in inset) FE-SEM images of the Li deposition obtained by plating 1 mAh cm^{-2} Li on Cu substrate at 0.1 mA cm^{-2} in Li||Cu cells using **a** 1 M $LiPF_6$ -FEC: FEMC and **b** 1 M $LiPF_6$ -FEC: FEMC: HTE electrolytes. Scale bars: 5 µm in Supplementary Figure 19a, b; 10 µm in the inset of Supplementary Figure 19a, b.

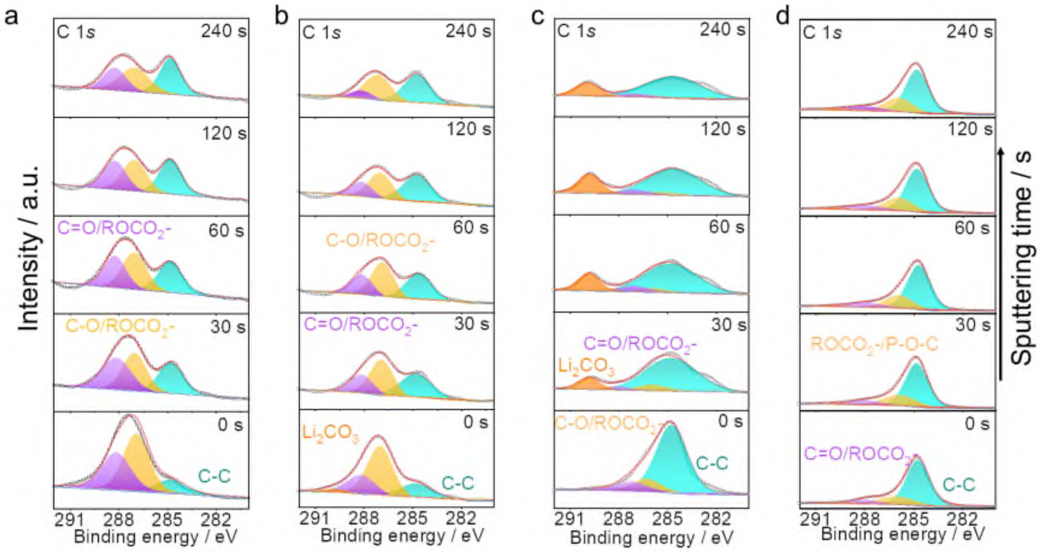

Supplementary Figure 20 C 1*s* in-depth XPS spectra of the Cu substrate obtained from **a** Li|1 M $LiPF_6$-EC: EMC|Cu, **b** Li|1 M $LiPF_6$-FEC: FEMC|Cu, **c** Li|1 M $LiPF_6$-FEC: FEMC: HTE|Cu and **d** Li|HGPE|Cu cells after 10 cycles. During the cycling, 1 mAh cm^{-2} Li was repeatedly plated on-stripped off the Cu foil at 0.2 mA cm^{-2}. C-C: 284.8 eV, C-O: 286.5 eV; C=O:288 eV[1]; Li_2CO_3:290 eV[2].

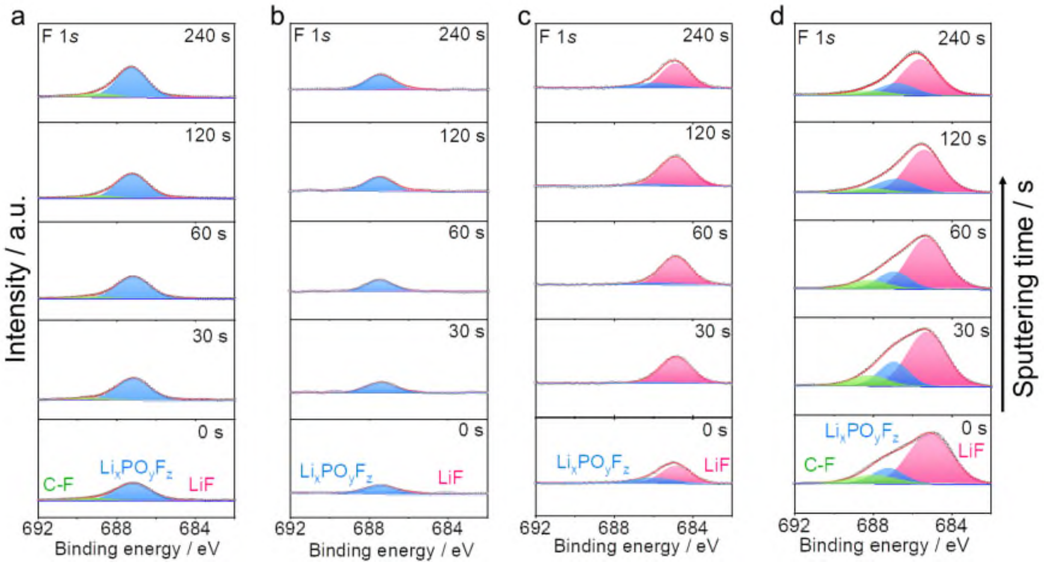

Supplementary Figure 21 F 1*s* in-depth XPS spectra of the Cu substrate obtained from **a** Li|1 M $LiPF_6$-EC: EMC|Cu, **b** Li|1 M $LiPF_6$-FEC: FEMC|Cu, **c** Li|1 M $LiPF_6$-FEC: FEMC: HTE|Cu and **d** Li|HGPE|Cu cells after 10 cycles. During the cycling, 1 mAh cm^{-2} Li was repeatedly plated on-stripped off the Cu foil at 0.2 mA cm^{-2} C-F: 688 eV; LiF: 685 eV[3]; $Li_xPO_yF_z$: 687 eV[4].

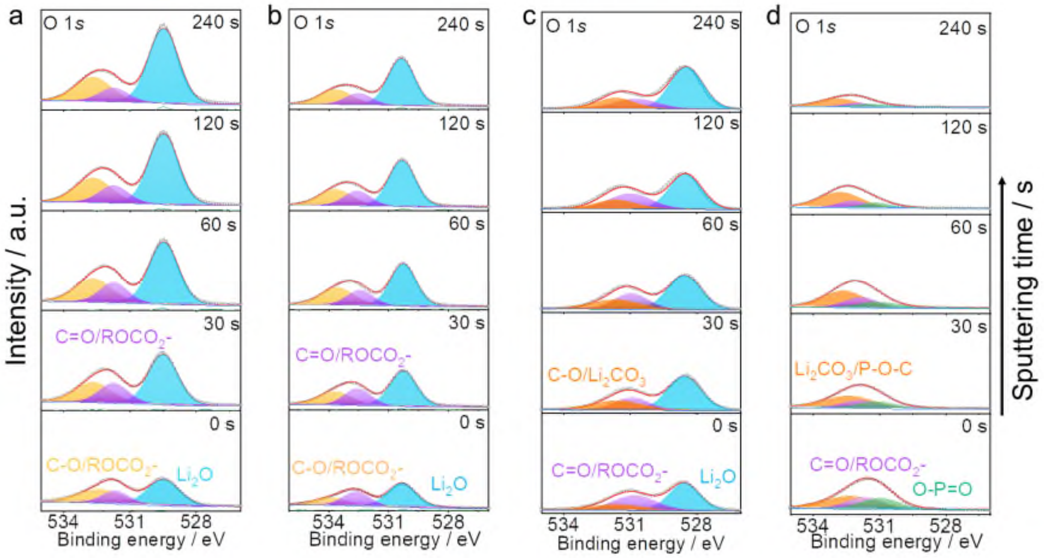

Supplementary Figure 22 O 1*s* in-depth XPS spectra of the Cu substrate obtained from **a** Li|1 M $LiPF_6$-EC: EMC|Cu, **b** Li|1 M $LiPF_6$-FEC: FEMC|Cu, **c** Li|1 M $LiPF_6$-FEC: FEMC: HTE|Cu and **d** Li|HGPE|Cu cells after 10 cycles. During the cycling, 1 mAh cm^{-2} Li was repeatedly plated on-stripped off the Cu foil at 0.2 mA cm^{-2} Li_2O: 529 eV[5]; C=O: 531.5 eV[2];C-O=533 eV[6]; Li_2CO_3: 532 eV[5]; P-O: 532.5 eV[7]; O-P=O: 531 eV[8].

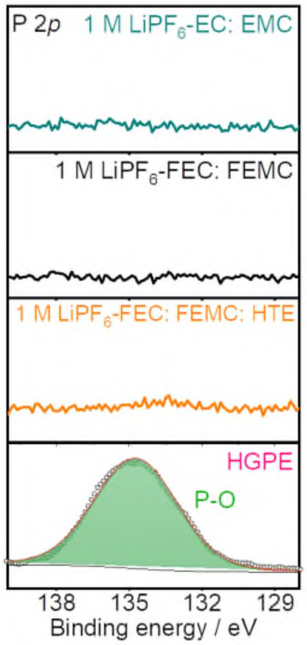

Supplementary Figure 23 The P 2*p* XPS spectra of the Cu substrate obtained from Li||Cu cells after 10 cycles. P-O: 135 eV[9].

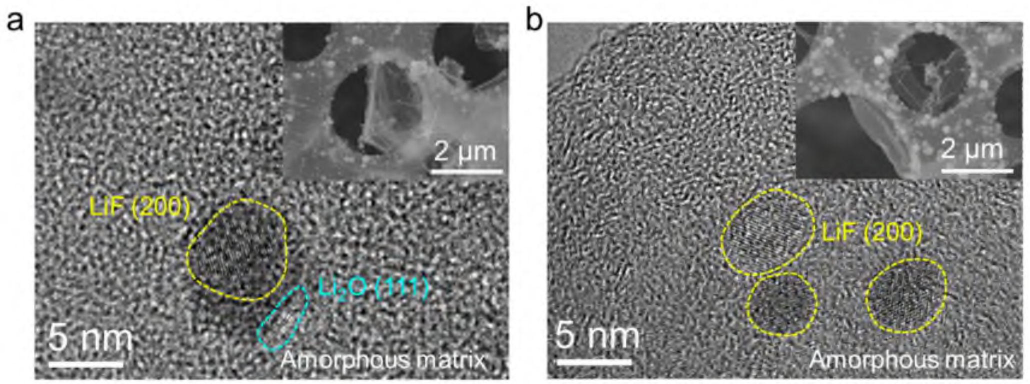

Supplementary Figure 24 TEM images of the SEI shell formed by plating/stripping Li on Cu grids in **a** 1 M $LiPF_6$-FEC: FEMC and **b** 1 M $LiPF_6$-FEC: FEMC: HTE electrolytes. Scale bars: 5 nm in Supplementary Figure 24a, b; 2 μm in the insets of Supplementary Figure 24a, b.

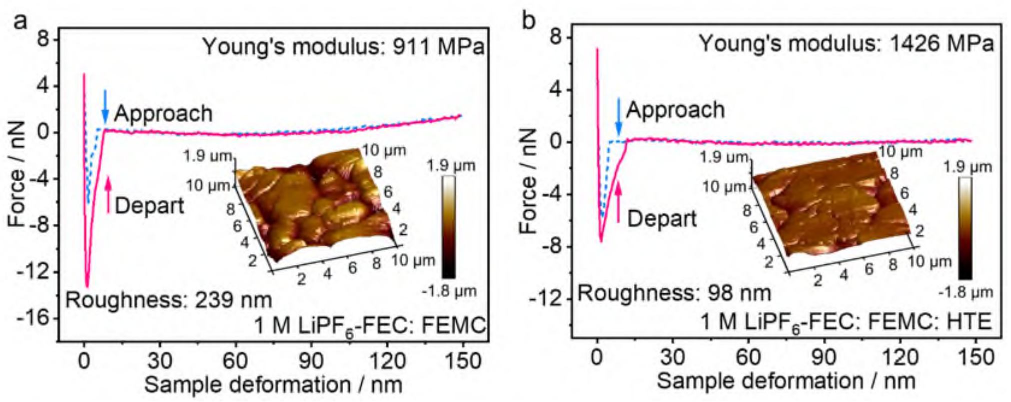

Supplementary Figure 25 Force-displacement plots of **a** 1 M $LiPF_6$ -FEC: FEMC derived SEI and **b** 1 M $LiPF_6$ -FEC: FEMC: HTE derived SEI. The corresponding 3D AFM scanning images of SEI layers are shown in insets.

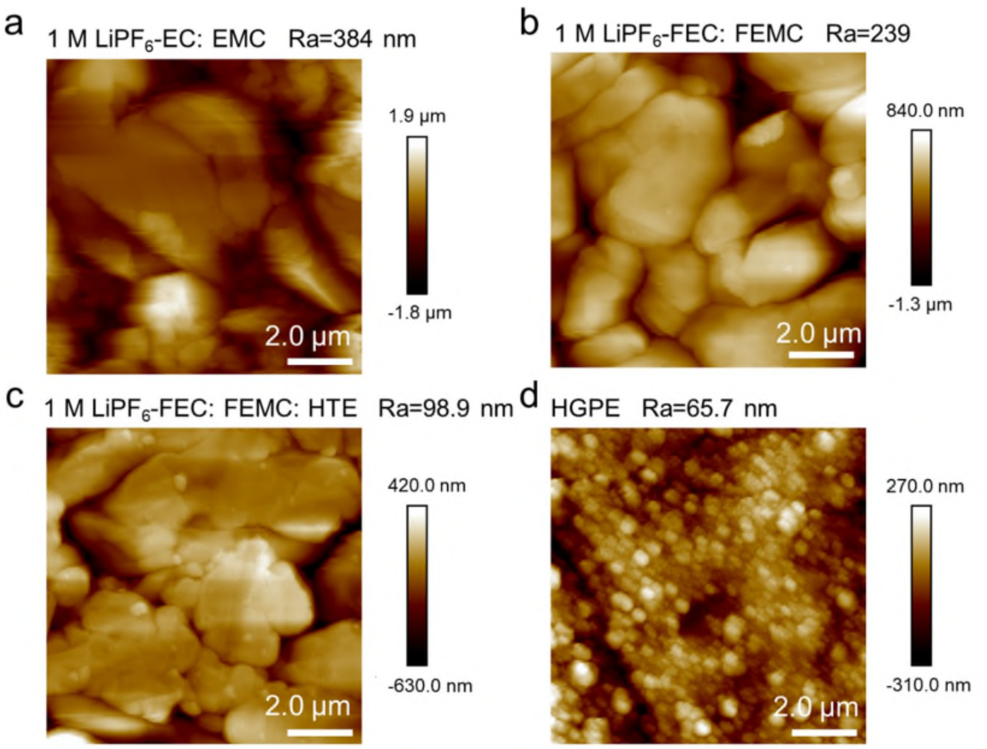

Supplementary Figure 26 Two-dimensional (2D) AFM images of **a** 1 M $LiPF_6$ -EC: EMC derived SEI, **b** 1 M $LiPF_6$ -FEC: FEMC derived SEI, **c** 1 M $LiPF_6$ -FEC: FEMC: HTE derived SEI and **d** HGPE derived SEI. The roughnesses are 384 nm, 239 nm, 98.9 nm and 65.7 nm, respectively. Scale bars: 2.0 μm in Supplementary Figure 26a-d.

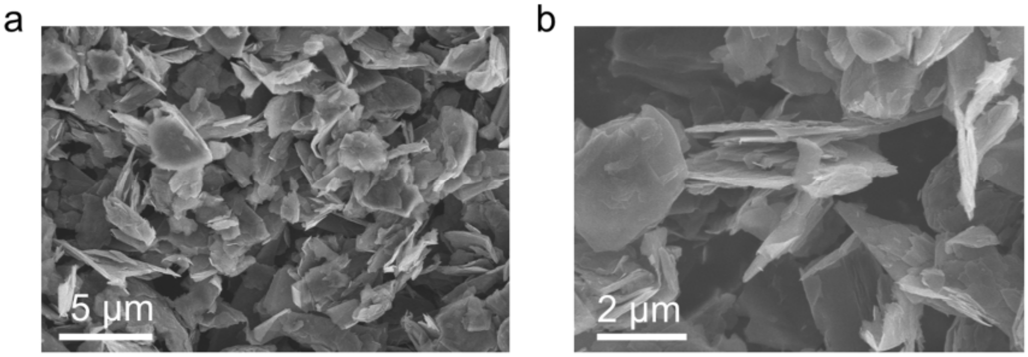

Supplementary Figure 27 SEM of KS6 graphite particle. Scale bars: 5 μm in Supplementary Figure 27a; 2 μm in Supplementary Figure 27b.

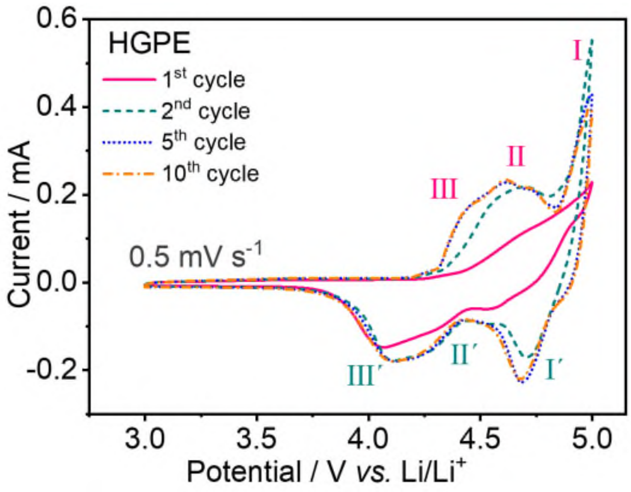

Supplementary Figure 28 CV curves of the Li|HGPE|KS6 graphite cell. The scan rate was 0.5 mV s^{-1}. The battery was aged for one day before testing. We speculate the activation process in the 1st cycle is mainly due to insufficient wettability of HGPE to electrodes.

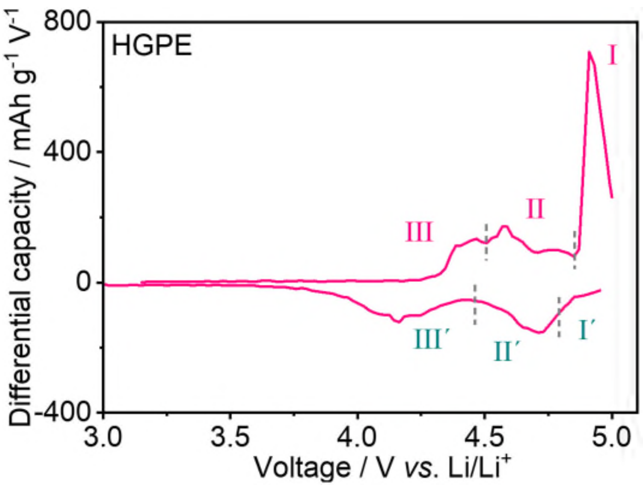

Supplementary Figure 29 dQ/dV curve of the Li|HGPE|KS6 graphite cell (Supplementary Note 3).

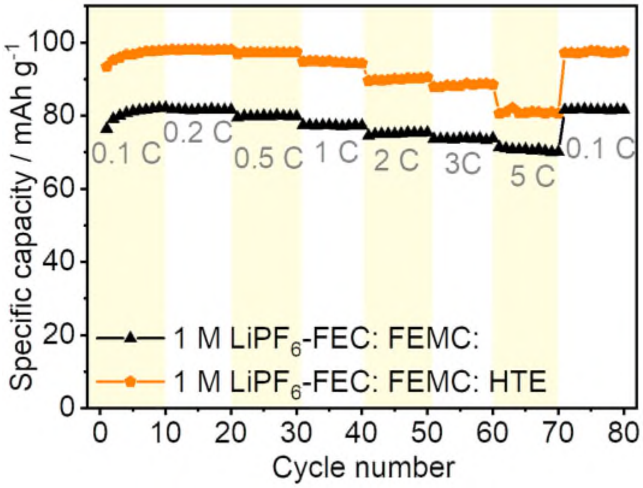

Supplementary Figure 30 Rate performances of Li||KS6 graphite cells employing 1 M $LiPF_6$-FEC: FEMC and 1 M $LiPF_6$-FEC: FEMC: HTE electrolytes under different current densities.

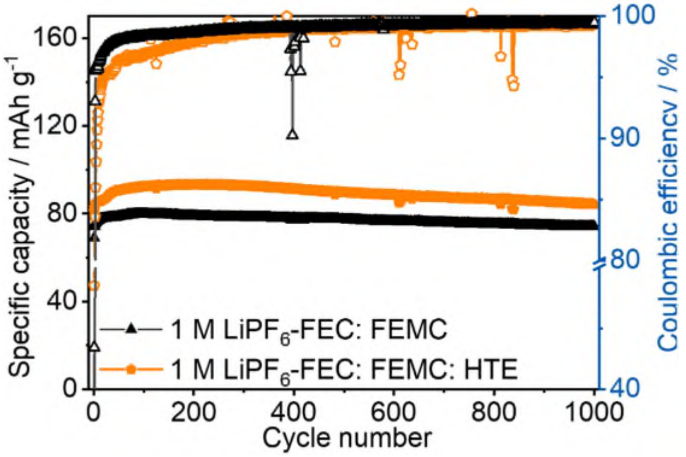

Supplementary Figure 31 Long-term cycling performance Li||KS6 graphite DIBs at 100 mA g^{-1} using 1 M $LiPF_6$-FEC: FEMC and 1 M $LiPF_6$-FEC: FEMC: HTE electrolytes.

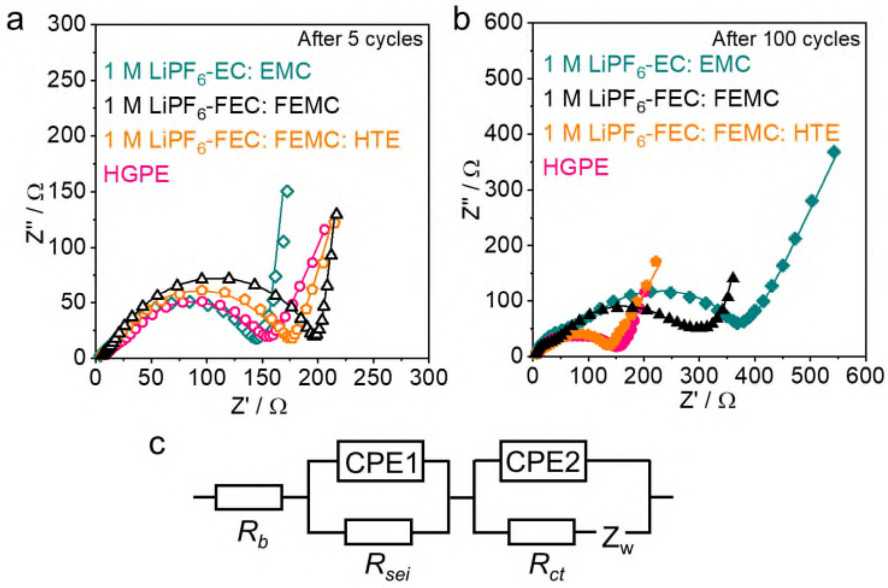

Supplementary Figure 32 a b EIS spectra of the Li||KS6 graphite cells using four electrolyte samples after **a** 5 cycles and **b** 100 cycles at 100 mA g^{-1}. **c** The corresponding equivalent circuit diagram. The errors between the raw and fitted EIS data were less than 2 % (Supplementary Note 4).

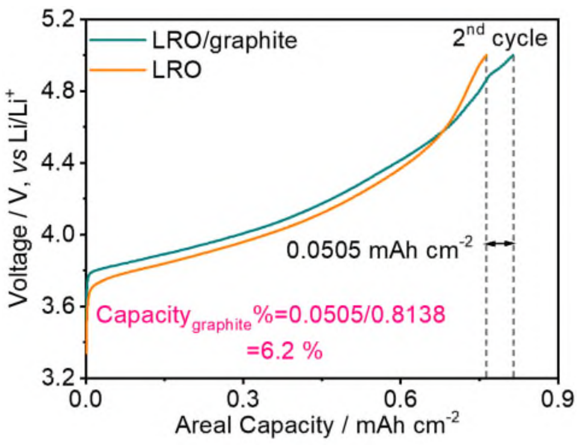

Supplementary Figure 33 Estimation of the capacity contribution of KS6 graphite (Supplementary Note 5).

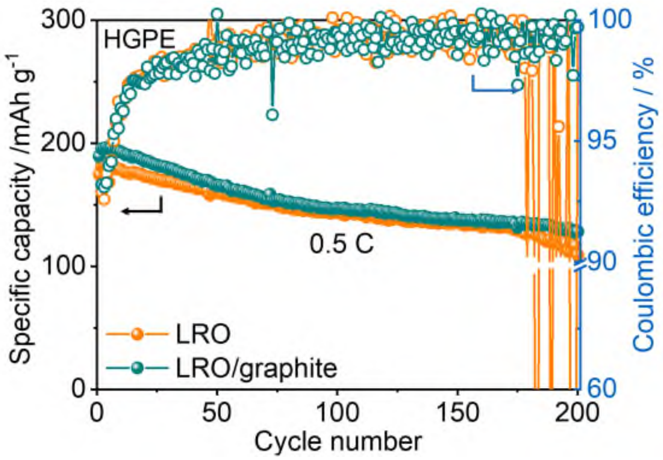

Supplementary Figure 34 Cyclic performances of Li|HGPE|LRO and Li|HGPE|LRO/graphite cells at 0.5 C.

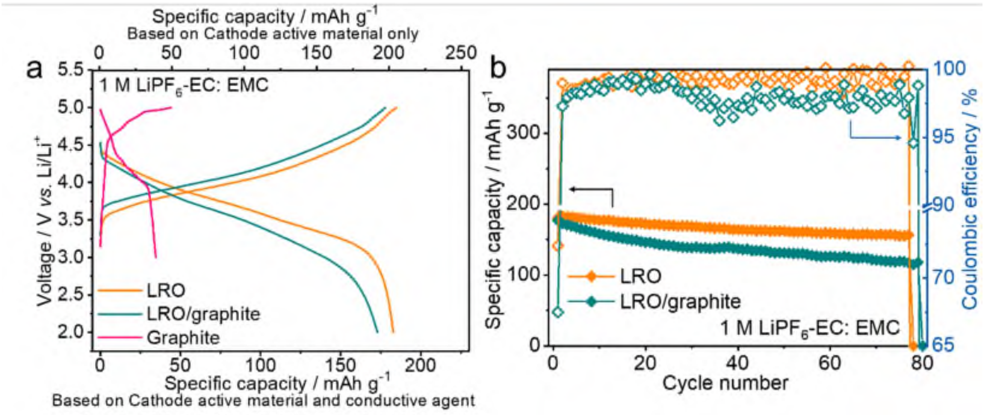

Supplementary Figure 35 a Charge-discharge curves of the Li||KS6 graphite, Li||LRO, and Li||LRO/graphite hybrid cells using 1 M $LiPF_6$-EC: EMC electrolyte at 0.2 C. **b** Cyclic performances of Li||LRO and Li||LRO/graphite hybrid cells using 1 M $LiPF_6$-EC: EMC electrolyte at 0.2 C. The capacity is calculated based the total mass of LRO and conductive agent.

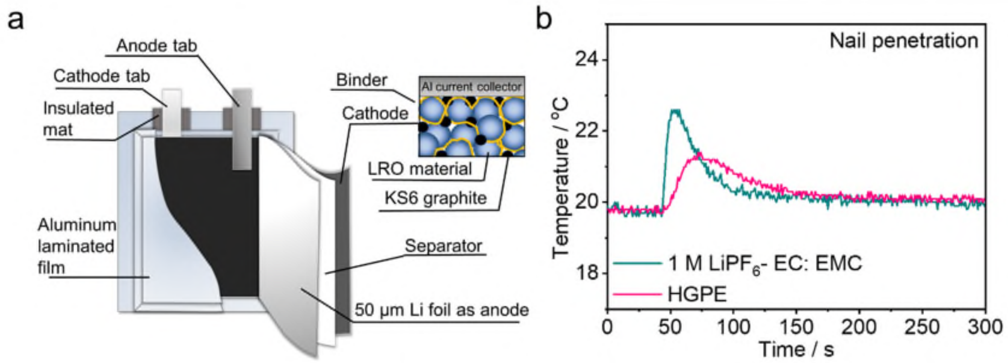

Supplementary Figure 36 a Schematic illustration of a SRLMB pouch cell configuration. **b** Temperature changes of fully charged Li|1 M $LiPF_6$-EC: EMC|LRO and Li|HGPE|LRO pouch cells during nail penetration tests.

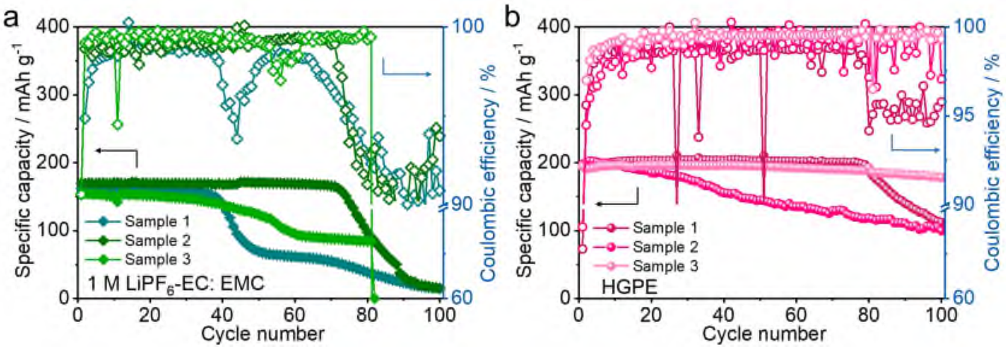

Supplementary Figure 37 a Cycling performances of Li||LRO/graphite pouch cells using **a** 1 M $LiPF_6$-EC: EMC electrolyte and **b** HGPE at 0.2 C.

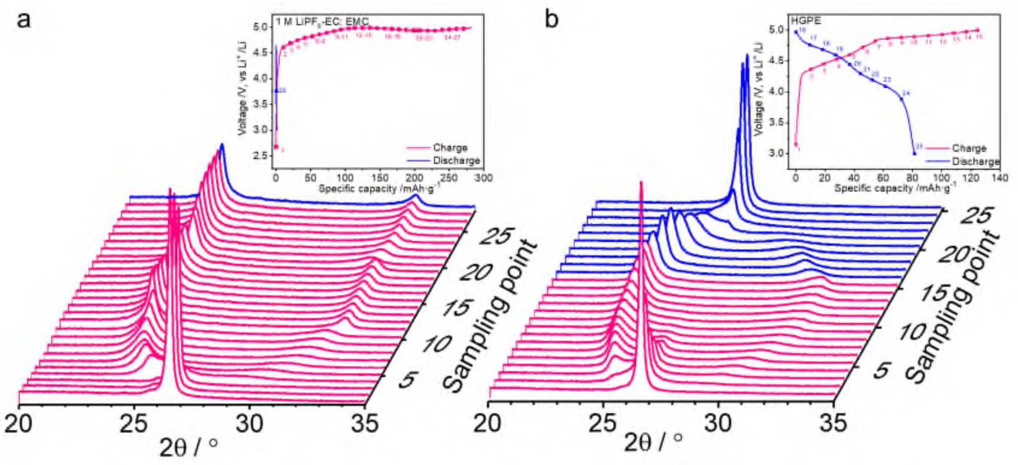

Supplementary Figure 38 *In situ* XRD patterns of the graphite cathode in **a** Li|1 M $LiPF_6$-EC: EMC|KS6 graphite and **b** Li|HGPE|KS6 graphite cells during the initial charge-discharge processes at 0.05 C. The numbers labeled in the charge-discharge profiles in the insets correspond to the ordinate values in the XRD patterns.

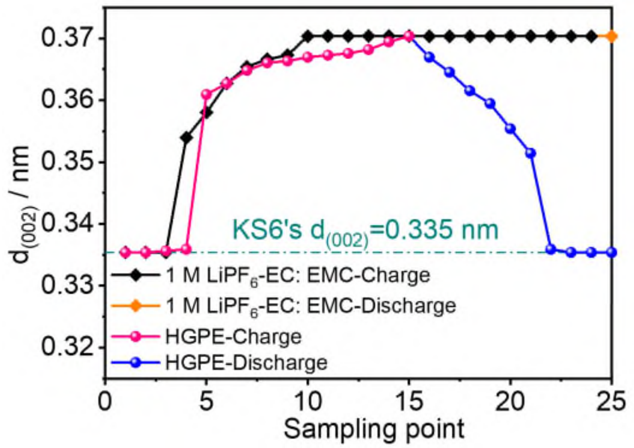

Supplementary Figure 39 The calculated $d_{(002)}$ interplanar spacings of the KS6 graphite cathode materials. The results were obtained from the *in situ* XRD patterns of Li|1 M $LiPF_6$-EC: EMC|KS6 graphite and Li|HGPE|KS6 graphite cells during the initial charge-discharge processes.

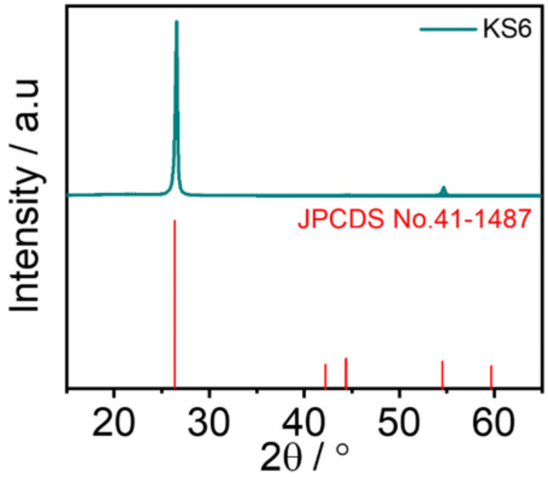

Supplementary Figure 40 The XRD pattern of pristine KS6 graphite powders.

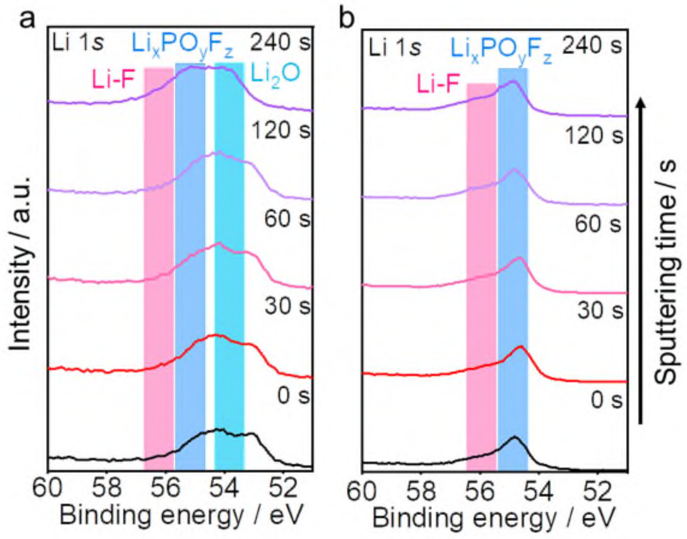

Supplementary Figure 41 Li 1*s* XPS depth spectra of in KS6 graphite cathodes obtained from **a** Li|1 M $LiPF_6$-EC: EMC|KS6 graphite and **b** Li|HGPE|KS6 graphite cells graphite after one cycle at 0.05 C.

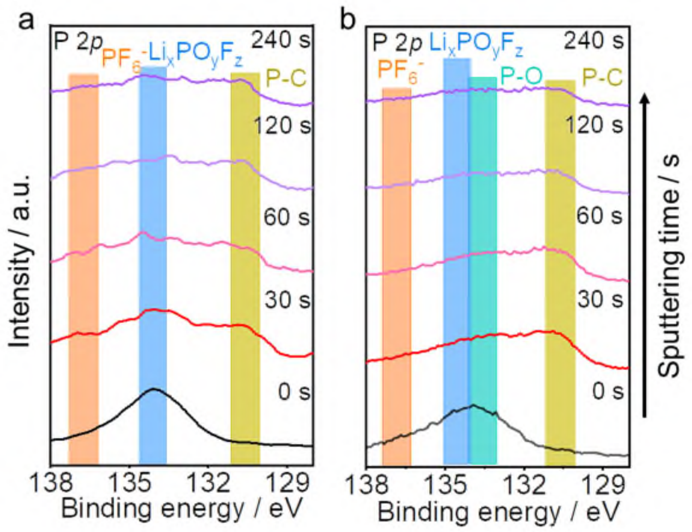

Supplementary Figure 42 P 2*p* XPS depth spectra of the KS6 graphite cathodes obtained from **a** Li|1 M $LiPF_6$-EC: EMC|KS6 graphite and **b** Li|HGPE|KS6 graphite cells after one cycle at 0.05 C.

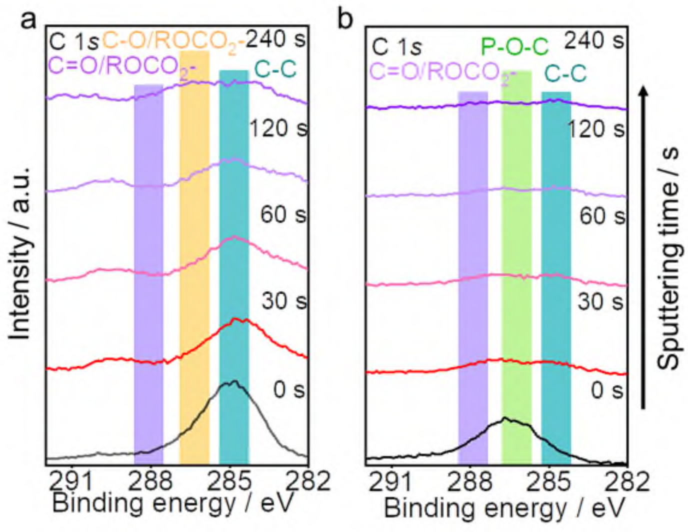

Supplementary Figure 43 C 1*s* XPS depth spectra of the KS6 graphite cathodes obtained from **a** Li|1 M $LiPF_6$-EC: EMC|KS6 graphite and **b** Li|HGPE|KS6 graphite cells after one cycle at 0.05 C.

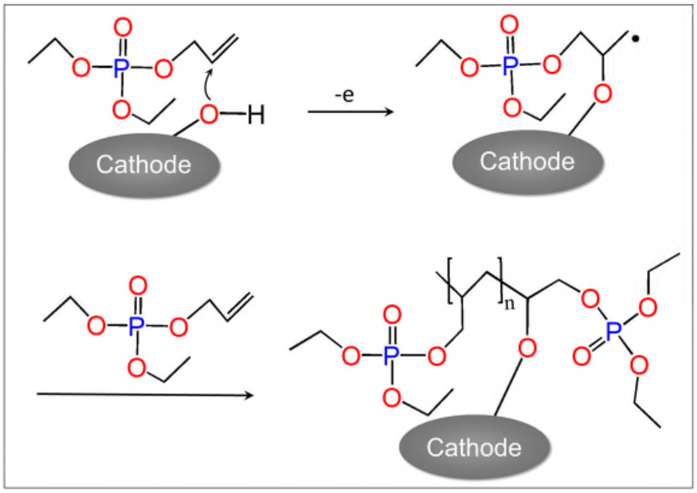

Supplementary Figure 44. A possible electro polymerization mechanism of residual DAP monomer on the graphite surface.

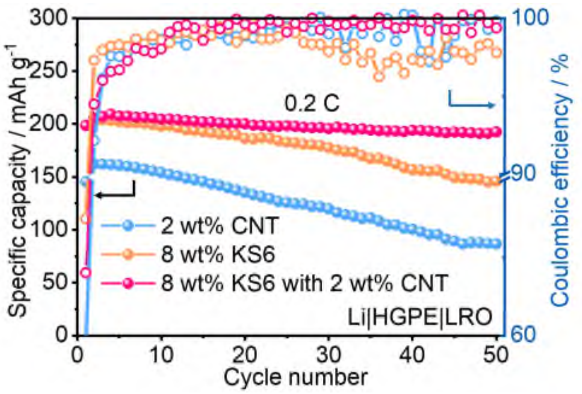

Supplementary Figure 45 Cyclic performances of Li|HGPE|LRO cells with 2 wt% CNT, 8 wt% KS6 graphite, and 2 wt% CNT with 8 wt% KS6 graphite at 0.2 C. We used two kinds of carbon as conductive agents in the cells, i.e., 8 wt% KS6 graphite and 2 wt% CNT. KS6 graphite was applied to support the "shuttle-relay" chemistry, while CNT was introduced to optimize the electronic conductive network in the cells since the dispersibility of KS6 graphite is relative poorer than tradition conductive agents (e.g., acetylene black) due to its higher tap density. As shown in Supplementary Figure 47, we compared the performance of Li|HGPE|LRO cells with 2 wt% CNT only, 8 wt% KS6 graphite only and 2 wt% CNT with 8 wt% KS6 graphite as conductive agents, respectively. Only the cell using 2 wt% CNT with 8 wt% KS6 graphite exhibited a stable cycling. This clearly demonstrated that the synergistic effect of KS6 graphite and a small amount of CNT facilitated the construction of a fast and robust electronic conductive network.

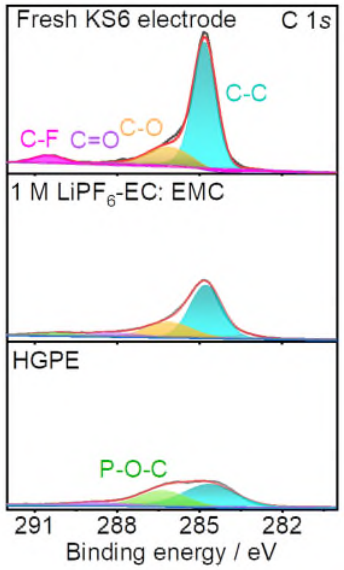

Supplementary Figure 46 The C 1*s* XPS spectra of fresh KS6 graphite electrode and cycled KS6 graphite electrodes in various electrolytes (Supplementary Note 6).

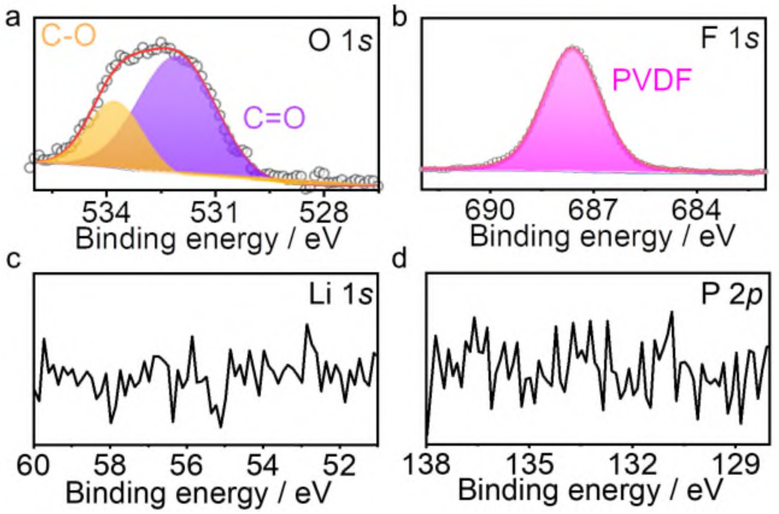

Supplementary Figure 47 The **a** O 1*s*, **b** F 1*s*, **c** Li 1*s* and **d** P 2*p* XPS spectra of fresh KS6 graphite electrode (Supplementary Note 6).

Supplementary Tables

Supplementary Table 1 The ionic conductivity values at 25 ºC and VTF fitting parameters of the four electrolyte samples.

**Electrolytes**	**$\sigma_{\circ}$ at 25 ºC ($S\ cm^{-1}$)**	**$\sigma_{\circ}$ ($S\ cm^{-1}\ K^{-1/2}$)**	**E_a (eV)**	**$T_{\circ}$ (K)**
1 M $LiPF_6$-EC: EMC	7.47×10^{-3}	0.711	6.95×10^{-3}	193.76
1 M $LiPF_6$-FEC: FEMC	2.92×10^{-3}	0.381	7.46×10^{-3}	199.08
1 M $LiPF_6$-FEC: FEMC: HTE	2.05×10^{-3}	0.372	9.30×10^{-3}	191.90
HGPE	1.99×10^{-3}	0.351	1.00×10^{-2}	183.27

Supplementary Table 2 The t_{Li}^+ values of different electrolytes (Supplementary Figure 14).

Electrolytes	R_b^0	R_i^0	R_b^{SS}	R_i^{SS}	I^0	I^{SS}	t_{Li}^+
1 M $LiPF_6$-EC: EMC	2.85	36.6	3.02	39.2	0.24	0.16	0.22
1 M $LiPF_6$-FEC: FEMC	2.22	59.3	2.33	70.9	0.188	0.10	0.21
1 M $LiPF_6$-FEC: FEMC: HTE	2.79	29.7	2.96	35.3	0.277	0.19	0.37
HGPE	8.83	154.2	9.16	176.18	0.048	0.033	0.43

Supplementary Table 3 Fitted values of the impedance spectra in a Li|1 M $LiPF_6$-EC: EMC |Li cell (Supplementary Figure 16b).

T(K)	1000/T(1/K^{-1})	R_{sei}	Ln(T/R_{sei})	R_{ct}	Ln(T/R_{ct})
283	3.533569	4.482	4.145378	35.7	2.070296
293	3.412969	2.432	4.791459	16.38	2.884112
303	3.30033	1.078	5.638625	8.434	3.581462
313	3.194888	0.546	6.351339	4.622	4.215376
323	3.095975	0.06848	8.458866	2.755	4.764235

Supplementary Table 4 Fitted values of the impedance spectra in a Li|1 M $LiPF_6$-FEC: FEMC|Li cell (Supplementary Figure 16c).

T (K)	1000/T (1/K^{-1})	R_{sei}	Ln(T/R_{sei})	R_{ct}	Ln(T/R_{ct})
283	3.533569	15.29	-1.46489	203.5	-4.05336
293	3.412969	10.27	-1.10164	114.1	-3.50949
303	3.30033	4.686	-0.35056	34.81	-2.35588
313	3.194888	2.17	0.386825	16.24	-1.62592
323	3.095975	0.99	1.140155	7.416	-0.87354

Supplementary Table 5 Fitted values of the impedance spectra in a Li|1 M $LiPF_6$-FEC: FEMC: HTE|Li cell (Supplementary Figure 16d).

T (K)	1000/T (1/K^{-1})	R_{sei}	Ln(T/R_{sei})	R_{ct}	Ln(T/R_{ct})
283	3.533569	1279	-5.89153	380.6	-4.67944
293	3.412969	573.2	-5.12365	58.12	-2.83493
303	3.30033	230.1	-4.24449	35.79	-2.38365
313	3.194888	98.81	-3.43165	17.29	-1.68858
323	3.095975	50.77	-2.7972	6.164	-0.68862

Supplementary Table 6 Fitted values of the impedance spectra in a Li|HGPE|Li cell (Supplementary Figure 16e).

**T (K)**	**1000/T (1/K^{-1})**	R_{sei}	**Ln(T/R_{sei})**	R_{ct}	**Ln(T/R_{ct})**
283	3.533569	328.9	-0.15031	237.1	0.176965
293	3.412969	250.2	0.157912	122.6	0.871246
303	3.30033	91.76	1.194556	53.88	1.726973
313	3.194888	45.45	1.92959	25.85	2.493893
323	3.095975	28.72	2.420059	13.13	3.202753

Supplementary Table 7 The EIS simulation results of the Li||KS6 graphite cells in the four electrolyte samples. The results are obtained from the four electrolyte samples after selected cycles corresponding to Supplementary Figure 32.

**Electrolytes**	R_{sei} **at 5th**	R_{sei} **at 100th**	R_{ct} **at 5th**	R_{ct} **at 100th**
1 M $LiPF_6$-EC: EMC	30.2	79.9	100.5	297.7
1 M $LiPF_6$-FEC: FEMC	40	46.7	156.8	212.5
1 M $LiPF_6$-FEC: FEMC: HTE	38.7	35.8	113.7	77.1
HGPE	42.9	16.11	80	125.7

Supplementary Notes

Supplementary Note 1. The influence of salt concentration on the ionic conductivity of electrolyte and battery performance.

The anions intercalation/deintercalation into/from graphite has little effect on the overall electrolyte concentration by calculating the concentration change of PF_6^- anions during the initial and final stages of charge/ discharge processes. The calculation process is as follows: take conductive graphite||Li battery as an example, the mass loadings of the active materials on each electrode were ≈1.0 mg cm^{-2} and the area capacity was 0.121 mAh cm^{-2}=0.438 C=2.73*10^{18} e, consumed n(e)=n(PF_6^-)=2.73*10^{18} e/6.02*10^{23}=4.53*10^{-6} mol, c(PF_6^-)=4.53*10^{-6} mol/6*10^{-5} L=0.0756 mol L^{-1} and the residual c(PF_6^-)=0.924 mol L^{-1}.

We have calculated the concentration change of PF_6^- anions during the initial and final stages of charge /discharge processes of Li|HGPE|LRO/graphite cells. The salt concentrations in electrolyte were 1 M and 0.92 M before and after PF_6^- intercalation into graphite. As shown in Supplementary Figure 1a, the ionic conductivity slightly increased from 1.99 to 2.05 mS cm^{-1} at 25 ^oC during this process. Furthermore, it is well-known that the salt concentration could dramatically affect the performance of dual-ion batteries based on anion shuttling[10]. As seen from Supplementary Figure 1, when the $LiPF_6$ concentration was set as 0.5 M in the HGPE (labeled as “0.5 M-HGPE”), the Li||LRO/graphite cell showed poor cycling performance (Supplementary Figure 1c) with large polarization (Supplementary Figure 1b) although the electrolyte ionic conductivity was as high as 2.61 mS cm^{-1} at 25 ^oC (Supplementary Figure 1a), mainly due to the insufficient salt concentration to support the anion intercalation. When the slat concentration reached 1.5 M in the HGPE, the ionic conductivity sharply reduced to 0.49 mS cm^{-1} at 25 ^oC owing to formation of excess ion pairs (Supplementary Figure 1a), which deteriorated the cycling performance (Supplementary Figure 1c) and caused the increase of the battery polarization (Supplementary Figure 1b). Therefore, the slat concentration was optimized as 1 M in the HGPE in this work, which exhibited the

highest cycling stability (Supplementary Figure 1c) with the lowest polarization (Supplementary Figure 1b).

Supplementary Note 2. The conversion rates of monomers in HGPE.

We investigated the conversion rates of DAP and PETEA monomers by ^{1}H NMR spectra[11]. The conversion rate of monomers can be estimated from the integrated area ratio of C$\boldsymbol{H_2}$= on the monomers in the polymerized gel/solution to that in pristine precursor solution. The C$\boldsymbol{H_2}$- on FEMC solvent was set as reference. As shown in Supplementary Figure 6a-f, after a polymerization at 70 °C for 60 min, the conversion rates of 3 wt% DAP only and 1.5 wt% PETEA only in the liquid electrolyte (1 M $LiPF_6$-FEC: FEMC: HTE (1:6:3 by volume)) containing 0.1 wt% AIBN initiator were 15.6 % (Supplementary Figure 6a and b) and 90.0 % (Supplementary Figure 6c and d), respectively. Notably, after heating the precursor solution containing 3 wt% DAP and 1.5 wt% PETEA, the conversion rate of DAP increased to 32.4 % and that of PETEA decreased to 81.9 % in the HGPE, indicating a copolymerization of these two monomers (Supplementary Figure 6e and f). Moreover, by varying the monomer ratio in the AIBN-containing electrolyte (the total monomer amount was set as 4.5 wt%), the competitive polymerization rate can be obtained following the formulas below[12]:

$$\frac{\mathrm{d}[M_{DAP}]}{\mathrm{d}[M_{PETEA}]}=\frac{[M_{DAP}](r_{DAP}[M_{DAP}]+[M_{PETEA}])}{[M_{PETEA}](r_{PETEA}[M_{PETEA}]+[M_{DAP}])} \quad \text{(S1)}$$

Where the r_{DAP} and r_{PETEA} is the competitive polymerization rate of two monomers; $[M_{DAP}]$ and $[M_{PETEA}]$ are the concentrations of two monomer; $\frac{d[M_{DAP}]}{d[M_{PETEA}]}$ is composition of copolymer, which is estimated by ratio the integral peak area of two monomers. We define $\rho=\frac{d[M_{DAP}]}{d[M_{PETEA}]}$ and $R=\frac{[M_{DAP}]}{[M_{PETEA}]}$, combing formula (S1) to obtain formula (S2)

$$R-\frac{R}{\rho}=\frac{R^2}{\rho}\mathrm{r}_{\mathrm{DAP}}-\mathrm{r}_{\mathrm{PETEA}} \quad \text{(S2)}$$

r_{DAP} and r_{PETEA} can be obtained from the slope and intercept of a (R-$\frac{R}{\rho}$)- $\frac{R^2}{\rho}$ plot, respectively. It is seen that r_{DAP} is 2.86 while r_{PETEA} is 0.06 (Supplementary Figure 6g-

h), suggesting that the DAP and PETEA monomers are copolymerized and the matrix of HGPE is a block copolymer rather than semi-interpenetrating polymer networks (sIPN)/ interpenetrating polymer networks (IPNs)[12, 13]. This can be further confirmed by the polymerization phenomenon in Supplementary Figure 6i, in which the gelation time of 1.5 wt% PETEA with 3 wt% DAP in liquid electrolyte is shorter than that of the 1.5 wt% PETEA in liquid electrolyte, demonstrating a copolymerization of these two monomers.

Supplementary Note 3. Analysis on the dQ/dV and CV results.

A dQ/dV differential curve of the Li|HGPE|KS6 graphite cell is shown in Supplementary Figure 29. Peaks in the profile correspond to electrochemical processes in the HGPE-based DIB during charging-discharging (Figure 4a). Stage III contains a broad weak peak, while stage II contains a strong peak and a small shoulder peak, consistent with previous reports[14]. The strong peaks in stage I represent both the formation of the corresponding graphite intercalation compound and the construction of the CEI film during the initial charging process. Such distinct identification of the different voltage stages in the cycling process indicates that PF_6^- anions successfully intercalate into the graphite layer, resulting in a generation of stage phases of graphite. Moreover, the de-intercalation peak is in agreement with the intercalation peak, with a potential downshift of 0.1-0.3 V, indicating a reversible electrochemical behavior. Accordingly, three pairs of redox peaks are also observed in the CV curve of the Li|HGPE|graphite cell (Supplementary Figure 28), corresponding to the three main processes of the anion intercalation in graphite.

Supplementary Note 4. EIS spectra of the Li||graphite cells using different electrolytes.

EIS measurements were performed to evaluate the interfacial characteristics and reversibility of the cells employing these different electrolytes. Figure 5c and Supplementary Figure 32a and b show the EIS results of Li||KS6 graphite cells with various electrolytes after different cycles, which were simulated by the equivalent

circuit shown in Supplementary Figure 32c. The corresponding simulation results are summarized in Supplementary Table 7.

According to the equivalent circuit in Supplementary Figure 32c, the intersection with the real axis refers to a bulk resistance (R_b) that reflects the resistance of the electrodes and the electrolyte/separator.The depressed semicircle at high frequencies can be ascribed to the SEI (R_{sei}) and *CPE1*, while the depressed semicircle at medium frequency can be attributed to the charge transfer resistance (R_{ct}) and *CPE2*. Instead of interfacial capacitors (C_f) and double-layer capacitors (C_{dl}), *CPE1* and *CPE2* are normal-phase elements used to account for the roughness of the particle surface. The line at low frequencies corresponds to the Warburg impedance (Z_w), which is associated with the diffusion of Li ions within the particle[15].

It is seen that the R_{sei} of the cell with 1 M $LiPF_6$-EC: EMC electrolyte increased dramatically from 30.2 Ω in the 5th cycle to 79.9 Ω in the 100th cycle. This can be attributed to the thick CEI and unstable SEI layers resulted from severe electrolyte decomposition on the cathode and Li dendrite growth on the anode, respectively (Figure 5c and Supplementary Figure 32a and b). The significantly increased R_{ct} (from 100.5 Ω to 297.7 Ω) can be ascribed to the high barrier of electron/ion transport in the electrode|electrolyte interface, resulting in the irreversibility of batteries. Notably, after the introduction of fluorinated solvents, the values of R_{sei} and R_{ct} was significantly reduced after cycling, probably due to the stabilized CEI/SEI layers and the enhanced electron/ion transport in the electrode|electrolyte interface[16, 17]. Moreover, it is seen that the value changes of R_{ct} and R_{sei} in the Li|HGPE|KS6 graphite cells is relatively small during cycling (R_{sei}: from 42.9 Ω to 16.1 Ω; R_{ct}: from 80.0 Ω to 125.7 Ω. Figure 5c and Supplementary Figure 32a and b). Such high stability of the electrode|HGPE interface facilitates the cycling performance improvement in Figure 5c.

Supplementary Note 5. Estimation of the areal capacity and energy density contribution of KS6 graphite in SRLMBs.

We calculated the areal capacity and energy density contribution of KS6 graphite based on the charging curve of Li|HGPE|LRO/KS6 graphite pouch cell. The areal capacity can be obtained from the following formula:

$$\text{Areal capacity}=\frac{Q}{A} \tag{S3}$$

where Q represents the capacity of cells and A represents the area of cathodes. As shown in Supplementary Figure 33, the areal capacity of the Li||LRO/KS6 graphite and Li||LRO cells were 0.8138 mAh cm^{-2} and 0.7633 mAh cm^{-2}, respectively. The areal capacity originating from the anion intercalation between the graphite layers was approximately equivalent to the areal capacity gap between the Li||LRO/KS6 graphite and Li||LRO cells, i.e., 0.0505 mAh cm^{-2}.

Furthermore, the energy density can be calculated from the formula below:

$$\text{Energy density}=\frac{Q\times E}{V} \tag{S4}$$

where Q represents the capacity of cells, E represents the average voltage of cells and V represents the volume of pouch cell. Considering the average voltage of Li||LRO/KS6 and Li||LRO graphite cells were 4.12 V and 4.04 V, respectively, the energy densities of Li||LRO/KS6 graphite and Li||LRO cells were 39.2 Wh L^{-1} and 36.0 Wh L^{-1}. The energy density delivered by the anion insertion graphite layer was approximately equivalent to the energy density gap between the Li||LRO/KS6 graphite and Li||LRO cells, i.e., 3.2 Wh L^{-1}. Moreover, the areal capacity and energy density contribution ratios of KS6 graphite can be estimated as:

$$\text{Capacity contribution}_{\text{graphite}}=\frac{0.0505}{0.8138}\times 100\ \%=6.2\ \% \tag{S5}$$

$$\text{Energy density contribution}_{\text{graphite}}=\frac{3.2}{39.2}\times 100\ \%=8.2\ \% \tag{S6}$$

Supplementary Note 6. The XPS analysis of fresh KS6 graphite cathode.

As seen in the C1s spectrum of the fresh KS6 graphite cathode in Supplementary Figure 46, the peaks at 286.0 eV and 288.0 eV are associated with the PVDF binder (consistent with the peaks at 531.4 eV and 533 eV attributed to C=O and C-O in the O1s spectrum,

and the peak at 687.5 eV ascribed to PVDF in the F 1s spectrum of the fresh graphite electrode, Supplementary Figure 47), while the C=C peak at 284.8 eV is attributed to the sp2 hybridization in the graphite structure[18].

Supplementary references

1. He, M. et al. High voltage $LiNi_{0.5}Mn_{0.3}Co_{0.2}O_2$/graphite cell cycled at 4.6 V with a FEC/HFDEC-based electrolyte. *Adv. Energy Mater.* **7**, 1700109 (2017).
2. Fan, X, et al. Non-flammable electrolyte enables Li-metal batteries with aggressive cathode chemistries. *Nat. Nanotechnol.* **13**, 715-722 (2018).
3. Jiang, L. -L. et al. Inhibiting solvent co-intercalation in a graphite anode by a localized high-concentration electrolyte in fast-charging batteries. *Angew. Chem. Int. Ed.* **60**, 3402-3406 (2020).
4. Li, W. -H. et al. Highly improved cycling stability of anion de-/intercalation in the graphite cathode for dual-ion batteries. *Adv. Mater.* **31**, 1804766 (2019).
5. Chen, J. et al. Electrolyte design for LiF-rich solid–electrolyte interfaces to enable high-performance microsized alloy anodes for batteries. *Nat. Energy* **5**, 386-397 (2020).
6. Zhang, Y. et al. Solvent molecule cooperation enhancing lithium metal battery performance at both electrodes. *Angew. Chem. Int. Ed.* **59**, 7797-7802 (2020).
7. Xia, J. et al. Study of triallyl phosphate as an electrolyte additive for high voltage lithium-ion cells. *J. Power Sources* **295**, 203-211 (2015).
8. Zhu, X. et al. Black phosphorus revisited: a missing metal-free elemental photocatalyst for visible light hydrogen evolution. *Adv. Mater.* **29**, 1605776 (2017).
9. Wang, Z. et al. Phosphorus-doped reduced graphene oxide as an electrocatalyst counter electrode in dye-sensitized solar cells. *J.Power Sources* **263**, 246-251 (2014).
10. Placke, T. et al. Perspective on performance, cost, and technical challenges for practical dual-ion batteries. *Joule* **2**, 2528-2550 (2018).
11. Zhao, Q. et al. Solid-state polymer electrolytes with in-built fast interfacial transport for secondary lithium batteries. *Nat. Energy* **4**, 365-373 (2019).
12. Creutz, S. et al. Living anionic homopolymerization and block copolymerization of (dimethylamino)ethyl methacrylate. *Macromolecules* **30**, 6-9 (1997).
13. Kim, J. -M. et al. Multifunctional semi-interpenetrating polymer network-nanoencapsulated cathode materials for high-performance lithium-ion batteries. *Sci. Rep.* **4**, 4602 (2014).
14. Wang, G. et al. Self-activating, capacitive anion intercalation enables high-power graphite

cathodes. *Adv. Mater.* **30**, 1800533 (2018).

15. Liu, M. et al. Novel gel polymer electrolyte for high-performance lithium–sulfur batteries. *Nano Energy* **22**, 278-289 (2016).

16. von Aspern, N. et al. Fluorine and lithium: ideal partners for high-performance rechargeable battery electrolytes. *Angew. Chem. Int. Ed*. **58**, 15978-16000 (2019).

17. Piao, N. et al. Countersolvent electrolytes for lithium-metal batteries. *Adv. Energy Mater.* **10**, 1903568 (2020).

18. Li, H. et al. Layer-by-layer assembly and uv photoreduction of graphene–polyoxometalate composite films for electronics. *J. Am. Chem. Soc.* **133**, 9423-9429 (2011).
