# Supplementary material for: A synergistic exploitation to produce high-voltage quasi-solid-state lithium metal batteries
Source: Nat Commun. 2021 Sep 30;12:5746. doi: 10.1038/s41467-021-26073-6 (PMC8484457; doi:10.1038/s41467-021-26073-6)
Supplement: Supplementary file 3 — Description of Additional Supplementary Files [file 41467_2021_26073_MOESM3_ESM.pdf]

<u>Description of Additional Supplementary Files</u>

Supplementary Movie 1: Combustion test of 1 M $LiPF_6$ -EC:EMC

Supplementary Movie 2: Combustion test of HGPE

Supplementary Movie 3: Combustion test of 1 M $LiPF_6$ -FEC:FEMC

Supplementary Movie 4: Combustion test of 1 M $LiPF_6$ -FEC:FEMC:THE

Supplementary Movie 5: Flexible test of pouch cell with 1 M $LiPF_6$-EC: EMC

Supplementary Movie 6: Flexible test of pouch cell with HGPE
